# A Comprehensive Review of Total Hip Arthroplasty Outcomes in Post-traumatic Hip Arthritis: Insights and Perspectives

**DOI:** 10.7759/cureus.56350

**Published:** 2024-03-17

**Authors:** Abhishek Choudhary, Gajanan Pisulkar, Shounak Taywade, Abhiram A Awasthi, Ankur Salwan

**Affiliations:** 1 Orthopaedics, Jawaharlal Nehru Medical College, Datta Meghe Institute of Higher Education and Research, Wardha, IND

**Keywords:** long-term durability, implant technology, patient satisfaction, surgical outcomes, total hip arthroplasty (tha), post-traumatic hip arthritis

## Abstract

Post-traumatic hip arthritis presents a challenging condition characterized by degenerative changes in the hip joint following traumatic injury. Total hip arthroplasty (THA) is a cornerstone in managing this condition, offering significant pain relief, functional improvement, and enhanced quality of life. This comprehensive review aims to synthesize existing literature to elucidate the outcomes of THA in post-traumatic hip arthritis, exploring factors influencing surgical success and identifying areas for further research. Key findings reveal favourable clinical outcomes associated with THA, though considerations such as patient characteristics, surgical techniques, and implant selection impact outcomes. Implications for clinical practice underscore the importance of tailored preoperative assessment and ongoing advancements in surgical approaches and implant technology. Furthermore, opportunities for future research lie in long-term durability studies, patient-reported outcomes assessment, and exploration of innovative surgical techniques. Overall, THA emerges as a promising intervention for post-traumatic hip arthritis, yet continual refinement through research and innovation remains imperative to optimize patient care in this population.

## Introduction and background

Post-traumatic hip arthritis (PTHA) refers to the degenerative joint disease that develops in the hip joint following a traumatic injury, such as a fracture or dislocation. This condition can result in pain, stiffness, and functional impairment due to the damage inflicted on the articular surfaces of the hip joint during the initial injury. The prevalence of PTHA varies depending on the population studied and the severity of trauma but is notably higher among individuals who have experienced significant hip trauma [1].

Total hip arthroplasty (THA) plays a crucial role in managing PTHA by providing effective pain relief, restoring function, and improving the quality of life for affected individuals. Unlike conservative treatments, such as medication or physical therapy, which may offer limited relief, THA offers a definitive solution by replacing the damaged hip joint with a prosthetic implant. This surgical intervention addresses both the structural damage caused by the trauma and the subsequent degenerative changes, offering long-term benefits to patients suffering from PTHA [2].

This review aims to comprehensively evaluate the outcomes of THA in the management of PTHA. By synthesizing existing literature and clinical evidence, this review aims to provide insights into the effectiveness of THA as a treatment modality for PTHA, identify factors influencing surgical outcomes, and highlight areas for future research and improvement in clinical practice.

## Review

Etiology and pathophysiology of PTHA

Causes of PTHA

PTHA arises from acute traumatic injuries to the hip joint, such as fractures or dislocations [3,4]. While any joint injury can trigger traumatic arthritis, dislocations and fractures are the most prevalent causes [5]. Trauma-induced irregularities in joint surfaces prompt friction between them, hastening cartilage wear. On average, 20-50% of individuals experiencing joint trauma may eventually develop post-traumatic arthritis (PTA) [4]. PTHA may manifest as either osteoarthritis or inflammatory arthritis [4]. Although PTA often resolves spontaneously within several months, certain cases can progress to become chronic [4]. The likelihood of PTHA onset escalates with age, the occurrence of multiple injuries, and elevated body weight [4].

Pathophysiological Mechanisms Involved

The pathophysiological mechanisms underlying post-traumatic osteoarthritis (PTOA) encompass a range of processes, including chondrocyte death, mitochondrial dysfunction, reactive oxygen species production, subchondral bone remodelling, inflammation, and cytokine release. Initial joint trauma triggers acute PTA, which can lead to chronic consequences such as osteoarthritis. During the acute phase, trauma causes structural damage, hemarthrosis, and chondrocyte death, prompting an inflammatory response characterized by elevated levels of cytokines such as IL-1, IL-6, and tumour necrosis factor α (TNFα). These cytokines promote cartilage matrix degradation while inhibiting proteoglycan synthesis [6-8]. Subsequently, the chronic phase involves ongoing remodelling processes within the cartilage and other joint tissues, ultimately culminating in osteoarthritis. Inflammatory responses persist, compromising the synthesis of a new matrix and contributing to subchondral bone sclerosis and osteophyte formation, which serve as diagnostic markers of the disease [7,8]. These pathogenetic mechanisms underscore the intricate interplay of cellular and molecular events that drive the development and progression of PTOA.

Factors Influencing Disease Progression

Several factors can influence a disease's progression, encompassing host, viral, environmental, and genetic factors. For instance, weather conditions significantly influence disease development, where moisture levels can impact the survival of propagules, thereby affecting both the initiation and severity of epidemics [9]. In the context of HIV infection, a multitude of factors, including immunological, virological, environmental, and genetic variables, contribute to disease progression [10]. In the case of PTA, the trauma inflicted on the joint can result in damage to the articular cartilage, weakening its integrity and paving the way for the development of PTA. This condition typically manifests two to five years after the initial joint injury and is characterized by symptoms such as joint swelling, pain, and instability [11]. Additionally, THA has emerged as an effective intervention for managing PTA after acetabular fractures. However, this procedure carries an elevated risk of complications, notably infection, with a reported total joint revision rate of 9.7% [12]. These findings underscore the multifaceted nature of disease progression, influenced by various factors, necessitating comprehensive approaches to diagnosis and management.

THA: overview and techniques

Definition and History of THA

THA is a surgical cornerstone involving the replacement of a damaged hip joint with an artificial implant to mitigate pain and enhance mobility. Widely regarded as one of the most successful orthopaedic procedures in contemporary practice, THA offers dependable outcomes for individuals grappling with end-stage degenerative hip osteoarthritis [13,14]. The roots of THA trace back to the late 1800s, marked by pioneering efforts such as those of Dr. Themistocles Gluck, who conducted initial animal experiments to explore joint replacement options. In a noteworthy milestone in 1890, Dr. Gluck documented one of his 14 total joint arthroplasties, which featured an ivory femoral head replacement in a human patient. Subsequent advancements led to the collaboration between Dr. Austin Moore and trauma surgeon Dr. Robert Judet in the 1940s, culminating in the development of the inaugural modern hip replacement comprising a metal femoral stem, a polyethene acetabular component, and acrylic bone cement [13]. Presently, THA represents a highly efficacious procedure, furnishing reliable outcomes for individuals grappling with advanced degenerative hip osteoarthritis. Technological innovations and refined surgical techniques have propelled its evolution, underscoring its pivotal role in modern orthopaedic practice [13].

Surgical Approaches and Techniques

THA represents a surgical intervention to replace a damaged hip joint with an artificial implant, effectively alleviating pain and enhancing mobility. THA's most frequently utilized surgical approaches are the posterior approach (PA), direct anterior approach (DAA), and direct lateral approach (LA) [15-17]. The choice of approach hinges upon various factors, encompassing surgeon preference, prior surgical incisions, patient factors such as obesity and risk for dislocation, implant selection, and the extent of joint deformity [18]. Predominantly employed in the United States and globally, the PA is the most common THA surgical technique [15]. Conversely, the DAA is gaining traction within the hip arthroplasty community, with proponents touting advantages such as the earlier restoration of gait kinematics and low dislocation rates [16]. Notably, the LA boasts the lowest dislocation rates, whereas the PA exhibits the lowest overall complication rates [17].

Preoperative computed tomography (CT) scans are recommended to evaluate joint space narrowing, osteophytes, subchondral sclerosis, and planned centre of hip rotation [16]. Mastery of anatomical understanding is paramount across all approaches to optimize visualization of the femoral and acetabular structures, mitigate complications, and enhance patient outcomes [16]. Moreover, the selection of approach for THA should be predicated upon surgeon proficiency and familiarity with the chosen approach, with evidence suggesting that the pros and cons of each approach tend to equalize within six weeks postoperatively [17].

Implant Options and Materials

Hip replacement implants comprise four main components, the stem, cup, ball, and liner, with the choice of materials for these components influenced by various factors such as the patient's age, the level of physical activity, and the surgeon's recommendation. Metal-on-plastic implants, utilizing cobalt-chromium or titanium, are renowned for their cost-effectiveness and extensive safety and durability track record. However, metal-on-metal implants, once popular, have declined in favour due to concerns surrounding metal debris and ion release [19-21]. Ceramic materials find widespread use in the head and liner components of hip replacement implants, prized for their durability and scratch-resistant properties. Ceramic heads, characterized by their hardness and smoothness, contribute to reduced polyethene wear. However, past iterations of ceramic-on-ceramic implants faced issues such as intolerable squeaking, necessitating surgical revision [19-21].

**Figure 1:**
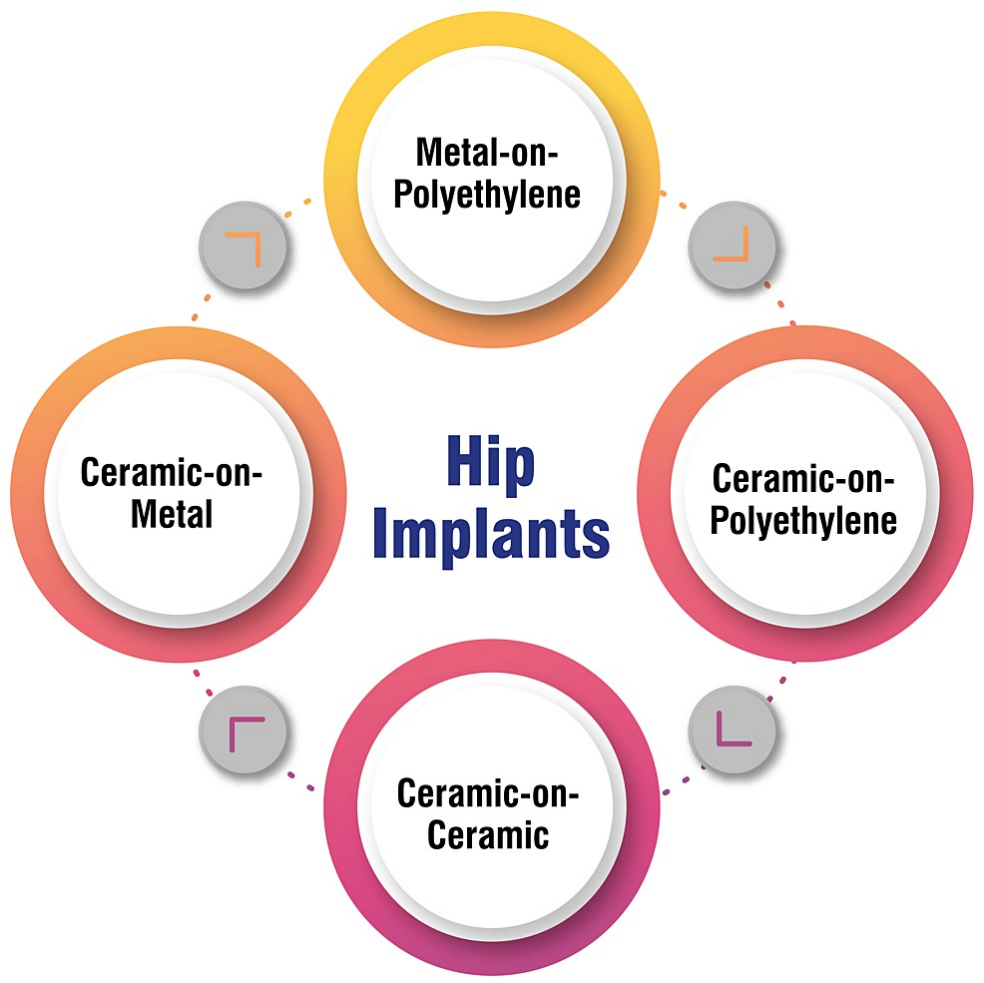
Implant options and materials The corresponding author self-created the figure

Plastic polyethene constitutes a crucial and widely utilized component in total hip replacement (THR). Historically associated with a notable risk of hip revision surgery due to wear, technological advancements have substantially mitigated these concerns over recent decades, reaffirming polyethene's importance in hip replacement implants [20,21]. The selection of implant material is a personalized decision influenced by patient-specific factors and the surgeon's expertise. Metal-on-plastic implants, with cobalt-chromium as the primary metal, remain the most prevalent choice for hip implants. Additionally, ceramic-on-polyethylene implants are commonly employed [20,21]. Therefore, thorough discussions with the surgeon are essential to understand the available options and weigh the pros and cons of each material, ensuring the best decision for the patient's unique circumstances. Implant options and materials are shown in Figure 1.

Outcomes of THA in PTHA

Clinical Outcomes

Pain relief: Recent research indicates that THA holds promise for reducing reported pain levels and enhancing functional outcomes in specific patient populations grappling with PTA following acetabular fractures [22]. The Harris Hip Score (HHS) emerged as the predominant scoring system across 18 studies encompassing 638 THA cases, revealing a mean preoperative score of 45, which significantly improved to 86 during follow-up assessments [22]. Nonetheless, it's crucial to acknowledge that THA for PTA entails heightened risks of complications compared to primary THAs, notably a 3.6% incidence of infection [22]. The total joint revision rate also stands at 9.7% [22]. Therefore, thoroughly considering potential benefits and risks is imperative before embarking on THA for cases of PTA stemming from acetabular fractures, necessitating transparent discussions about expectations with patients and caregivers [22].

Functional improvement: THA has emerged as a transformative intervention, yielding significant enhancements in functional outcomes for patients grappling with PTA secondary to acetabular fractures. The HHS is a prominent tool for assessing functional outcomes, with studies reporting a typical preoperative HHS of around 45, which substantially escalates to approximately 86 in postoperative assessments [4]. This signifies a noteworthy improvement in functional capacity among individuals undergoing THA for PTA. However, these favourable outcomes must be balanced against the heightened risk of complications, including infection and the need for revision surgeries [22].

Patient-reported outcomes: Investigations have delved into clinical outcomes, encompassing patient-reported outcomes after THA for various conditions, including PTA. Short-term assessments of patient-reported outcomes following THA have demonstrated improvements in pain and function [23]. Comparative studies between THA and hemiarthroplasty (HA) for hip fracture treatment have suggested superior outcomes with THA [24-26].

Radiographic Outcomes

Implant survivorship: Radiographic assessments concerning the survival of implants after THA in cases of PTHA primarily focus on the alignment and stability of the implanted components. Comparative studies between the DAA and the PA have revealed that the DAA is linked to higher rates of acceptable acetabular angles and a decreased likelihood of unacceptable inclination angles [27]. Furthermore, a systematic review and meta-analysis indicated no significant disparities between the DAA and PA regarding other radiographic outcomes, such as acetabular anteversion, limb length discrepancy, or offset [28]. Generally, maintaining appropriate positioning of acetabular and femoral components is paramount for ensuring implants' long-term success and durability. Proper alignment is critical in minimizing wear, loosening, and dislocation while promoting optimal biomechanics and function [27-29].

Complications: Radiographic evaluations following THA in the context of PTA and acetabular fractures have garnered significant attention. Radiographic assessments are pivotal in gauging the efficacy of hip arthroplasty, encompassing classifications based on various types and complications [30]. Complications arising after THA for PTA encompass prosthetic joint infection, with an observed incidence rate of 3.6% [22]. Notably, a systematic review comparing the DAA and PA in THA unveiled no notable differences in radiographic outcomes between the two approaches [28]. In a study scrutinizing clinical and radiographic outcomes of primary THA, consistent evidence of proximal bone ingrowth was discerned, with no complete radiolucent lines identified except around a loosened stem [29]. Radiographic assessments serve as indispensable tools for monitoring success and detecting potential complications after THA in patients grappling with PTA.

Patient Satisfaction and Quality of Life After THA

Information on radiographic outcomes, patient satisfaction, and quality of life following THA must be more extensive. However, recent research has shed light on the positive impact of THA on patient-reported outcomes, notably through metrics such as the HHS. Studies have indicated a notable increase in this score, from an average of approximately 45 to 86 points post-surgery [31,32]. Additionally, patient satisfaction with radiological services has been explored, with most respondents expressing contentment with the provided services [33]. Moreover, comparative investigations have highlighted the favourable outcomes of both THA and total knee arthroplasty (TKA) in terms of patient satisfaction and improvement in quality of life [34]. The Hip Disability and Osteoarthritis Outcome Score (HOOS) is a valuable tool in assessing hip function, encompassing pain, symptoms, activities of daily living, sports, and hip-related quality of life [35]. While further research is warranted to deepen our understanding of these outcomes, current evidence underscores the positive impact of THA on patient-reported measures and satisfaction levels.

Factors influencing THA outcomes

Patient-Related Factors

Age: Age is a pivotal patient-related factor impacting the outcomes of THA. Research indicates that optimal functional outcomes and prosthesis survival rates are often observed among patients aged 45-75, weighing less than 70 kg, possessing robust social support systems, boasting higher educational levels, exhibiting better preoperative functional statuses, and lacking significant comorbid diseases [36]. However, conflicting findings suggest that age alone may not significantly predict poor outcomes post-THA [37]. Therefore, it is crucial to contextualize age within the broader spectrum of patient-related factors, including sex, diagnosis, anxiety/depression, pain, and function, when assessing the potential outcomes of THA [37].

Comorbidities: Comorbidities represent patient-related factors that notably influence the outcomes of THA. Numerous studies have underscored the impact of conditions such as diabetes, chronic obstructive pulmonary disease (COPD), chronic kidney disease (CKD), and obesity in elevating the risk of postoperative complications, including periprosthetic joint infection and mortality [38,39]. Additionally, cardiovascular disease, hypertension, and depression have been associated with unfavourable outcomes following THA [40]. Accordingly, identifying patients with comorbidities preoperatively and managing them appropriately are crucial steps in mitigating the risk of complications and enhancing outcomes [38].

Body mass index (BMI): BMI represents a significant patient-related factor influencing the outcomes of THA. Research indicates that a notable proportion of THA patients, approximately 4.6%, present with obesity [39]. Furthermore, BMI has been identified as a factor affecting patient satisfaction post-THA, with higher BMI correlating with reduced satisfaction levels [37]. Obesity also heightens the risk of infection following primary hip replacement [39]. Therefore, considering BMI alongside other patient-related factors is imperative when evaluating the potential outcomes of THA, with preoperative discussions aimed at informing patients about the associated risks and benefits of the procedure.

Surgical Factors

Surgical approach: THA outcomes are influenced by various surgical factors, including anaesthesia, postoperative complications, and rehabilitation processes. Moreover, the choice of surgical approach can significantly impact outcomes. THA can be performed using different approaches, such as the DAA, PA, and LA, each offering distinct advantages and disadvantages [17]. Factors like accessibility, infection rates, and bone loss guide the selection of the surgical approach [41]. Additionally, considerations such as the type of prosthesis used, postoperative pain management, and psychological factors contribute to overall THA outcomes [42]. Careful evaluation of these factors is crucial during THA planning to optimize patient outcomes.

Implant selection: THA outcomes are influenced by nonsurgical and surgical factors. Nonsurgical factors encompass patient characteristics like gender, age, BMI, underlying health conditions, prosthetic material selection, and associated risk factors. Surgical factors, including anaesthesia, postoperative complications, and rehabilitation, also impact THA success [42]. Implant selection plays a pivotal role in determining THA outcomes, with considerations including the type of implant (cemented vs. uncemented), alignment, and fixation method. Choosing between cemented and uncemented implants can affect prosthesis longevity and stability, while proper alignment is essential for joint replacement function and durability. The fixation method, such as press-fit implants, may pose challenges in cases of poor bone quality [43,44].

Surgeon experience: THA outcomes are influenced by nonsurgical and surgical factors, with surgeon-related factors playing a significant role. Nonsurgical factors encompass patient characteristics and prosthetic material selection, while surgical factors include anaesthesia, postoperative complications, and rehabilitation processes [42]. Surgeon-related factors, particularly experience and technique, significantly impact hip replacement surgery outcomes [45]. Therefore, it is essential to consider these diverse factors comprehensively to optimize patient satisfaction and outcomes following THA.

Rehabilitation and Postoperative Care

**Figure 2:**
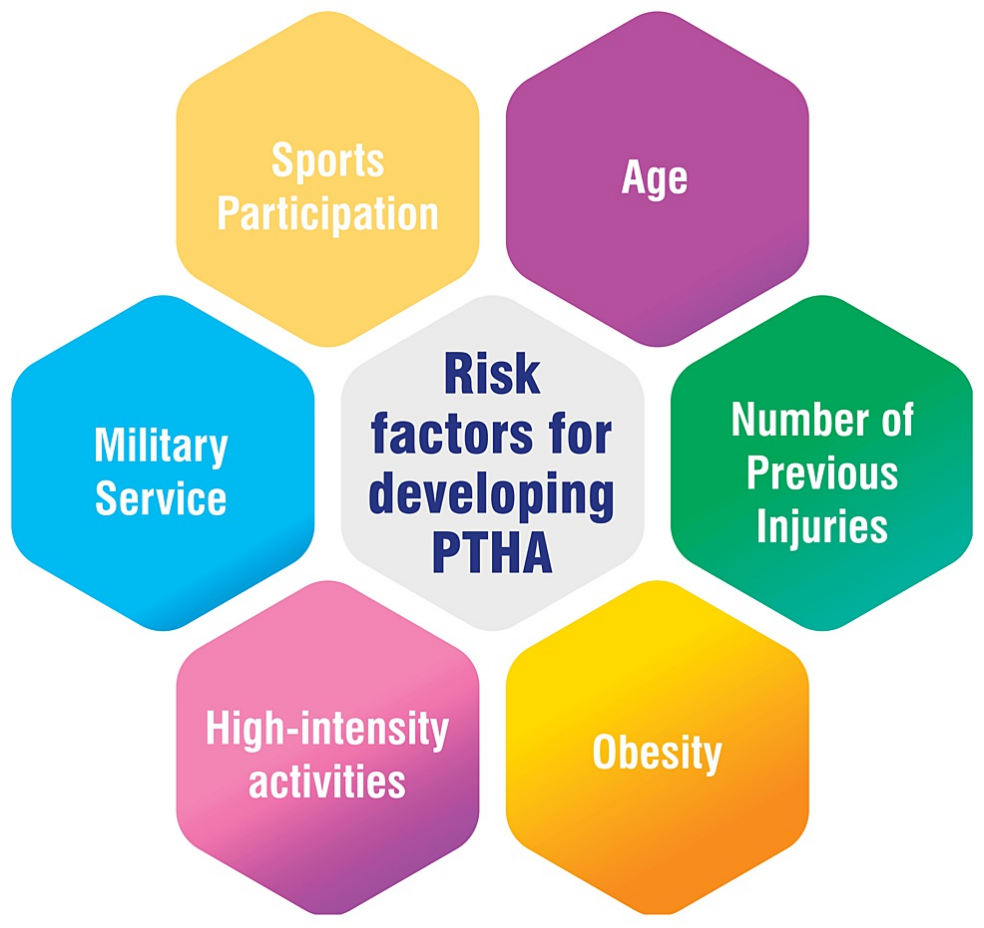
Risk factors for developing post-traumatic hip arthritis The corresponding author self-created the figure

Numerous factors contribute to THA outcomes, spanning nonsurgical and surgical realms. Nonsurgical factors encompass variables such as gender, age, BMI, choice of prosthetic material, and pertinent risk factors. Conversely, surgical factors encompass anaesthesia, postoperative complications, and rehabilitation [42]. Rehabilitation, a pivotal component of THA recovery, is critical in optimizing functional outcomes. Tailored immediate postoperative rehabilitation programs, accounting for individual patient characteristics such as gender, age, BMI, and diagnosis, wield significant influence over functional outcomes [46]. Moreover, variables like age, preoperative function, comorbidities, obesity, perioperative complications, prosthesis-related factors, postoperative pain management, and psychological factors exert notable impacts on postoperative recovery and functional outcomes after primary THA [46]. Thus, adopting a comprehensive approach that addresses these multifaceted factors is imperative to optimize the rehabilitation process and enhance overall functional outcomes for patients undergoing THA. Factors influencing THA outcomes are shown in Figure 2.

Challenges and complications in THA for PTHA

Bone Loss and Deformity

Bone loss and deformity pose significant challenges in THA, particularly in the context of PTHA. Young patients with severe hip damage or deformities, often stemming from conditions such as osteonecrosis or osteoarthritis, are increasingly undergoing hip replacement surgeries [47]. However, THA in young patients necessitates careful consideration due to concerns surrounding implant longevity, potential bone loss with future surgeries, and an elevated risk of complications [47]. To address these challenges, modern approaches in THA, such as cementless implants and advanced surgical techniques, are being employed to minimize bone loss during revision surgeries and enhance outcomes for younger patients [47]. In surgical planning, imaging studies like magnified X-rays, MRIs, and CT scans play a pivotal role in comprehensively evaluating joint angles, deformities, bone condition, and size. These imaging modalities aid in precise surgical planning, particularly in cases involving bone loss and deformity [47]. Moreover, a range of reconstructive options are available in revision THA procedures necessitated by femoral bone loss. These include impaction allografting, distal press-fit fixation, allograft-prosthesis composites, and the utilization of tumour mega prostheses, all aimed at addressing the extensive bone loss challenges encountered [48,49]. By implementing these advanced techniques and interventions, surgeons strive to overcome the complexities associated with bone loss and deformity, optimizing outcomes for patients undergoing THA.

Infection Risk

**Figure 3:**
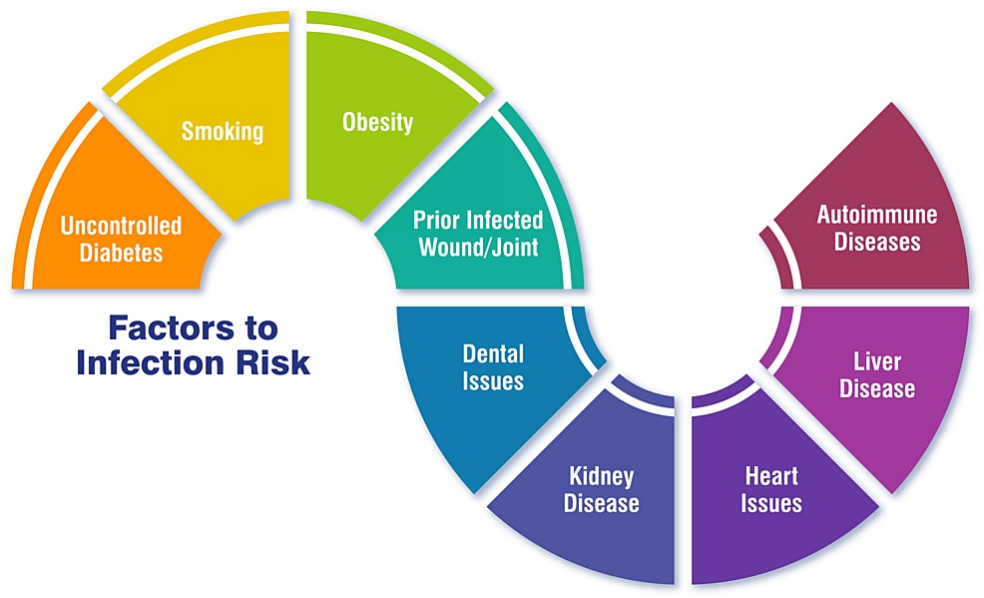
Factors of infection risk to post-traumatic hip arthritis The corresponding author self-created the figure

Infection risk poses a significant concern in THA procedures, with the incidence of infection after hip or knee replacement surgery typically ranging between 1% and 2% [50]. Various factors contribute to an increased risk of infection following joint replacement surgery, including immune deficiencies, diabetes mellitus, and other chronic medical conditions [50,51]. Infections can manifest shortly after surgery or even years later, with acute infections commonly occurring within four to six weeks post-surgery [50]. Preoperative optimization and identification of modifiable risk factors are paramount in mitigating the risk of surgical site infections in patients undergoing total joint arthroplasty. These risk factors may include obesity, anaemia, malnutrition, and diabetes mellitus [52]. Employing measures to prevent infections is crucial and may involve administering antibiotics before and after surgery, adhering to strict sterile techniques during the surgical procedure, conducting preoperative nasal screening for bacterial colonization, and implementing preoperative chlorhexidine wash procedures [51,52]. By addressing these risk factors and implementing preventive measures, healthcare providers aim to minimize the incidence of postoperative infections and optimize patient outcomes following THA procedures. Factors of infection risk are shown in Figure 3.

Dislocation

Dislocation is a potential complication following THR, with most occurrences manifesting within the initial months post-surgery during the healing phase of tissues [53,54]. Several factors can precipitate dislocation, including implant malposition, inadequate soft tissue tension, impingement, and patient noncompliance with postoperative precautions [53,55,56]. Depending on the underlying mechanical cause, dislocations can manifest in three directions: cranial, dorsal, and anterior [57]. The risk of dislocation escalates with age, a BMI exceeding 30 kg/m^2^, lumbosacral pathology, surgical experience, rheumatoid arthritis, and femoral head size [56]. Recalcitrant dislocations necessitate surgical intervention, which may involve trochanteric advancement, revision arthroplasty, or the utilization of constrained components [53,56]. Closed reduction of the hip constitutes the primary treatment modality for dislocation, while surgical management becomes imperative for irreducible dislocations, recurrent instability, and cases of implant malposition [53]. Post-dislocation physiotherapy adheres to the same protocol as total hip surgery, albeit with potential modifications tailored to the complexity of the procedure and the surgeon's specific recommendations [53]. Thus, a comprehensive approach encompassing preventive measures, prompt management, and tailored rehabilitation protocols is essential in mitigating the risk of dislocation and optimizing outcomes following THR.

Component Wear and Osteolysis

Component wear and osteolysis pose significant challenges in THA for PTHA, primarily attributed to the wear of ultra-high-molecular-weight polyethene (UHMWPE) [58,59]. Wear occurs through various modes, including fatigue, interfacial (adhesive and abrasive) wear, and third-body wear, leading to the deposition of wear particles in periprosthetic tissue and initiating osteolytic processes [58-60]. Factors such as pelvic orientation, vertical component positioning, and high volumetric polyethene wear contribute to wear and osteolysis [58]. Ongoing research aims to elucidate the intricate relationship between wear, osteolysis, and their subsequent consequences. Techniques for wear measurement, encompassing radiography and computer-aided methods, facilitate the tracking of wear patterns and inform decisions regarding revision surgery [58]. High-crosslink density UHMWPE has emerged as a promising avenue for reducing wear, although long-term data regarding its efficacy remain limited [59]. Continual efforts to advance materials and refine surgical techniques hold promise for enhancing THA's durability and longevity in managing PTHA.

Heterotopic Ossification (HO)

HO denotes the abnormal formation of mature lamellar bone within soft tissues where bone typically does not exist. It frequently arises after injuries or major surgeries, giving rise to bony growths that can elicit pain and restrict movement, particularly in proximity to joints [61,62]. While genetic HO is rare but tends to be more severe, lacking a definitive cure, non-genetic HO can often be managed utilizing nonsurgical treatments such as rest, ice therapy, and gentle stretching exercises [61,62]. Noteworthy risk factors for HO encompass a history of injuries or surgeries, with up to 75% of cases being associated with trauma [61,62]. Common triggers for HO include procedures like total hip or joint replacements, limb amputations, spinal cord injuries, and traumatic brain injuries [61,62]. The diagnosis of HO typically involves imaging modalities such as X-rays or CT scans, often utilizing grading scales like the Brooker classification to evaluate the extent of bone growth [61,62]. Treatment modalities for HO are contingent upon the severity of symptoms. They may encompass pain management strategies, administration of medications like corticosteroids, physical therapy aimed at preserving range of motion, and, in severe instances, surgical excision of mature bone growths [62]. Healthcare professionals endeavour to mitigate the adverse effects of HO and optimize patient outcomes by employing a multifaceted approach tailored to individual needs.

Future directions and innovations in THA

Technological advancements in THA have ushered in a new era of surgical precision and reproducibility. Integrating state-of-the-art robotic technology into THA procedures has notably facilitated more accurate acetabular positioning, potentially augmenting surgical precision and overall outcomes [63]. Innovative techniques have emerged to refine THA procedures further and optimize patient outcomes. Technologies such as virtual reality, three-dimensional printing, patient-specific instrumentation, and dual mobility bearings have been introduced, offering promising avenues for enhancing the efficacy of THA [63].

Looking toward the future, ongoing research endeavours in hip surgery are poised to revolutionize the field. Focus areas include investigating minimal invasiveness, developing enhanced recovery protocols, exploring newer technologies to improve outcomes, and establishing standardized approaches to revision THA surgery [64]. Despite the remarkable strides made in THA technology and technique, challenges and considerations persist. While advancements like robotic-assisted surgery offer improved planning and user experience, challenges such as higher costs and longer surgical times necessitate careful consideration for wider adoption and integration into clinical practice [63]. A patient-centric approach underscores the importance of tailoring THA procedures to meet patients' evolving needs and expectations, particularly as the demographic landscape shifts with a growing number of younger individuals undergoing THA. Optimizing functional outcomes, especially for those aspiring to return to sports activities, is paramount in this context [63].

## Conclusions

This review has synthesized critical findings regarding THA outcomes in managing PTHA. THA emerges as a valuable intervention, offering substantial pain relief, functional restoration, and enhanced quality of life for affected individuals. However, the review underscores the importance of considering various factors influencing surgical outcomes, including patient characteristics, surgical techniques, and implant selection. These insights have significant implications for clinical practice, advocating for a comprehensive preoperative assessment and ongoing advancements in surgical approaches and implant technology to optimize outcomes and minimize complications. Moreover, while THA represents a promising treatment modality, further research is warranted, particularly in long-term durability, patient-reported outcomes, and innovative surgical techniques, to continually refine and enhance the management of PTHA through THA. Overall, this review emphasizes the value of THA as a pivotal intervention in addressing the challenges posed by PTHA and underscores the ongoing need for research and innovation to improve patient outcomes in this population.
